# Beyond Being Insured: Insurance Coverage Denial as a Major Barrier to Accessing Care During Pregnancy and Postpartum

**DOI:** 10.1177/10547738231177332

**Published:** 2023-06-02

**Authors:** Jusung Lee, Krista J. Howard, Caleb Leong, Timothy J. Grigsby, Jeffrey T. Howard

**Affiliations:** 1 University of Texas at San Antonio, USA; 2 Texas State University, San Marcos, TX, USA; 3 University of Nevada, Las Vegas, USA

**Keywords:** delay in care, insurance coverage denial, financial constraints, transportation, rural

## Abstract

This study investigates the association between insurance coverage denial and delays in care during pregnancy and postpartum. An online survey was administered in March and April 2022 to women who were either pregnant or within 1 year postpartum (*n* = 1,113). The outcome was delayed care, measured at four time points: during pregnancy and 1 week, 2 to 6 weeks, and after 7 weeks postpartum. The key covariate was insurance coverage denial by providers during pregnancy. Delayed care due to having an unaccepted insurance and being “out-of-network” was more pronounced at 1 week postpartum with 3.37 times and 3.47 times greater odds and in 2 to 6 weeks postpartum with 5.74 times and 2.97 times greater odds, respectively. The association between insurance denial and delays in care encapsulated transportation, rural residency, time issues, and financial constraints. The findings suggest that coverage denial is associated with significant delays in care, providing practical implications for effective perinatal care.

## Introduction

The American College of Obstetricians and Gynecologists recommends that women receive comprehensive and thorough care during pregnancy and after childbirth (American Academy of Pediatrics and the American College of Obstetrics and Gynecologists, 2017; McKinney et al., 2018). Delaying care can entail substantial consequences, leaving untreated patients waiting for days or even opting for abandonment of recommended clinical tests (Robeznieks, 2018). A survey conducted by the American Medical Association underscores that a majority of physicians noted that delays in care are common (American Medical Association, 2018). The negative health impacts from delays are significant, including preterm birth, reduced birth weight, and maternal morbidity and mortality (Wilcox et al., 2016; Yeo et al., 2016). A lack of timely healthcare interventions can also lead to harmful or suboptimal health impacts among newborns as a delay in care is likely to elevate the risk of poor infant health outcomes, such as prevalent infant morbidity and mortality (Hollowell et al., 2011; Tayebi et al., 2013). Understanding the barriers to accessing care over the pregnancy trajectory is essential for promoting effective management of pregnancy and pregnancy-related health risks (Geiger et al., 2021; Kleppel et al., 2016).

Insurance coverage is an essential component for accessing health care in the United States. Over the past few years, the United States has achieved marked improvements in ensuring that Americans are insured; the passage of the *Affordable Care Act* (ACA), including Medicaid expansion, was a breakthrough to significantly reduce the uninsured rate, from 16.6% in 2013 to 11.0% in 2021 among Americans under age 65 (Lee et al., 2022). Despite the impressive progress through the historic health reform, accessing care continues to be a challenge due to barriers, such as high deductibles or out-of-pocket burden, a lack of time, or poor experiences with providers (Allen et al., 2017; Griffith et al., 2017; Kamimura et al., 2020; Kominski et al., 2017; Wolfe et al., 2020).

When reimbursement is not guaranteed or limited for the services provided, it can interrupt routine care proceedings and push providers to stop accepting a specific form of insurance coverage (Han et al, 2015). A prior authorization process is another structural barrier that can limit access to timely care (American Medical Association, 2018). Under conditions when administrative and clinical information offered by providers does not meet the approval requirements before the delivery of services, the precertification process can function as a substantial obstacle to receiving necessary medical examinations or medications (American Medical Association, 2022). Earlier reports have also suggested that patients often have low insurance literacy meaning they do not have sufficient knowledge of their insurance policy, including information about coverage restrictions and out-of-network providers, which pose practical barriers to seeking the needed care without delay (Levy & Janke, 2016; Loewenstein et al., 2013; Tipirneni et al., 2018).

Despite the potential role of insurance coverage denial in delaying care and escalating detrimental health impacts, little is known about the association of insurance coverage denial with a delay in care and the trends of their associations over the pregnancy trajectory. Also, there is little evidence to support the reasons why women experience delayed care during pregnancy and postpartum. To fill these gaps, we first investigated the association between insurance coverage denial and a delay in care during pregnancy and postpartum. We also examined the association between insurance coverage denial and specific reasons for delayed care.

## Materials and Methods

The data used in this study were collected from a cross-sectional, national survey of pregnant and postpartum women aged 18 and older, conducted from March 24, 2022 through April 23, 2022 (Howard et al., 2022). The study was reviewed and designated exempt by the University of Texas at San Antonio Institutional Review Board.

The participants in this study provided electronic consent by clicking their agreement to participate in the survey. The study included self-identified currently pregnant and postpartum women, up to 1 year after delivery, who were recruited and incentivized through national panels administered by Qualtrics. Qualtrics is a company that provides online survey tools as well as the management of national panels of survey respondents who have previously agreed to participate in surveys. Qualtrics panel members are recruited from various sources, including website recruitment ads, member referrals, targeted email lists, gaming sites, customer loyalty web portals, permission-based networks, and social media. Qualtrics performs panel identity verification prior to admission to their panel. Recruitment for our specific survey involved the sending of email survey invitations, sent by Qualtrics, to existing panel members, which ensured that responses were anonymous and deidentified to the researchers. Participants were provided a study information sheet in the email and the opportunity to provide consent and participate or decline. A total of 18,900 randomly selected individuals who self-identified as female were sent a survey invitation, and 2,739 (14.4%) participants clicked the link to provide consent and take the survey. Of the 2,739 individuals who clicked the survey link, 938 (34.2%) were excluded due to racial/ethnic strata quotas already being met. We used a 50% quota limit for non-Hispanic white women to ensure a sample with similar racial/ethnic composition as the total U.S. population of birth mothers. This process produced a sample with racial/ethnic composition similar to the total U.S. population of birth mothers, with 50% being non-Hispanic white and 50% be non-white mothers. In addition, 469 (17.1%) failed inclusion for refusing to provide consent or indicating they did not meet the current or recently pregnant criterion, 52 (1.9%) were excluded from the study due to data quality checks, and 1,280 (46.7%) participants completed the survey. The inclusion criteria of this study selected only those who reported delays in health care and those who reported any health insurance coverage, resulting in 1,171 participants. Among these, the analysis included 1,113 samples after excluding missing data (4.95%) on any variables in the study.

## Measures

### Key Outcomes

The key outcome was a delay in care during pregnancy and postpartum. The participants were asked the question, “Did you have a delay in care for any of the following reasons?” Respondents indicated whether or not the following items affected their experience of a delay in care at four different time points (during pregnancy, first week postpartum, 2–6 weeks postpartum, and after 7 weeks postpartum): did not have transportation, living in a rural area where the distance to the doctor is too far; nervous about seeing the doctor; could not get time off work; could not get childcare; could not afford the copay; could not afford the deductible; and could not afford to pay out of pocket for treatment. With responses to these items, binary variables were created for transportation, rural residence, and nervousness about seeing a doctor. A binary variable for financial constraints was constructed using could not afford the copay, could not afford the deductible, and could not afford to pay out of pocket for treatment. Also, a variable for a lack of time was dichotomized based on no access to childcare or could not get time off work. All of the dichotomized variables were also combined to create a single measure of any delay in care.

### Key Independent Variable

The key independent variable was insurance coverage denial by providers. Participants reported to a question, “During your current/recent pregnancy, were you ever told by your doctor or healthcare provider that they did not accept your healthcare coverage?” Response options were no, yes: provider did not accept, and yes: out of network.

### Secondary Covariates

We included demographic characteristics to adjust for potential confounding effects, such as age, race/ethnicity, health insurance, marital status, employment, and education (Feijen-de Jong et al., 2015). Age was categorized as 18 to 24, 25 to 35, 36 to 44, and 45 or above, and race/ethnicity was classified as Hispanic (any), or non-Hispanic Asian or Pacific Islanders, Black, White, and Other. Health insurance was coded as employer based, private, Medicaid, Medicare, and other. Marital status was coded as never married, married, or widowed/divorced/separated, and employment was categorized into unemployed, retired/student/housewife, part-time work, and full-time work. Education had six categories: some high school, high school diploma, some college, associate degree, bachelor’s degree, and graduate degree. Number of past live births (categorized as 0, 1, 2, and 3 or more) were included as secondary covariates of the pregnancy-related experience (McKenney et al., 2022; Vintzileos et al., 2002).

### Statistical Analysis

We presented sample characteristics using descriptive statistics. The percentage of any delay in care was calculated to describe the scope of delayed care from pregnancy up to 1 year postpartum. Furthermore, the percentage of a delay in care for specific reasons, such as financial constraints, transportation, nervousness, time issues, and rural residency, was calculated during pregnancy and postpartum. Given the primary interest of the study was the association between health insurance coverage denial by providers and a delay in care, a bivariate analysis was performed and presented to graphically assess the patterns of delayed care over time. We employed a multivariable logistic regression model to estimate the adjusted odds ratios (ORs), 95% confidence intervals (CIs), and *p* values. In the initial model, regression analysis was conducted to derive estimates for any delay in care during pregnancy and postpartum resulting from insurance coverage denial, adjusted for age, race/ethnicity, health insurance, marital status, employment, education, and a range of pregnancy-related past experiences. Subsequently, logistic regression models estimated ORs for a delay in care for a specific reason. All analyses were performed using R statistical software (version 4.1.3) and the statistical significance test was set to a two-tailed test with *p* < .05 indicating statistical significance.

## Results

Descriptive statistics (Table 1) show that 40.1% of the sample consisted of women aged 25 to 35, 29.6% were women between 36 and 44, 19.0% were aged 18 to 24, and 11.2% were 45 or above. The racial/ethnic breakdown of the participants in the sample was 43.3% Whites, 31.0% Hispanics, 15.0% Blacks, 6.2% Asian or Pacific Islanders, and 4.5% Others. The majority of the sample was covered by either employer based (37.6%) or government insurance coverage, such as Medicaid (26.7%) and Medicare (18.4%). Most participants reported one past live birth (36.8%), followed by those with two live births (25.9%), three or more live births (23.7%), and no past live births (13.6%). More than half of the sample had no experience of insurance coverage denial by providers, but 21.5% experienced unaccepted insurance and 21.4% experienced being out of network when accessing prenatal or postnatal care.

**Table 1.** Characteristics of the Study Sample (*N* = 1,113).

Factors	*N*	%
Age (years)		
18–24	212	19.0
25–35	446	40.1
36–44	330	29.7
≥45	125	11.2
Race		
Non-Hispanic White	482	43.3
Non-Hispanic Black	167	15.0
Hispanic	345	31.0
Non-Hispanic Asian/Pacific Islander	69	6.2
Non-Hispanic Other	50	4.5
Insurance		
Employer based	418	37.6
Private pay	149	13.4
Medicaid	297	26.7
Medicare	205	18.4
Other	44	3.9
Marital status		
Never married	296	26.6
Married	119	10.7
Separate/divorced/widowed	698	62.7
Employment		
Unemployed	134	12.0
Retired/student/housewife	110	9.9
Part time	199	17.9
Full time	670	60.2
Education		
Some high school	43	3.9
High school diploma	244	21.9
Some college	211	19.0
Associate degree	164	14.7
Bachelor’s degree	248	22.3
Graduate	203	18.2
Insurance denial		
No	636	57.1
Provider did not accept	239	21.5
Out of network	238	21.4
Previous live births		
0	151	13.6
1	410	36.8
2	288	25.9
≥3	264	23.7

**Figure 1.**
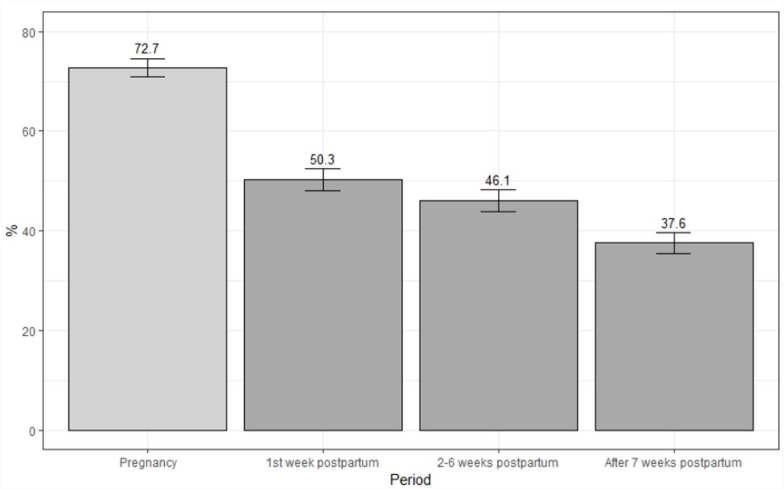
A delay in care during pregnancy through after 7 weeks postpartum. *Note.* Pregnancy and postpartum periods included 586 and 527 participants, respectively. The prevalence of delays in care was calculated individually for the pregnant women and women in their postpartum period.

**Figure 2.**
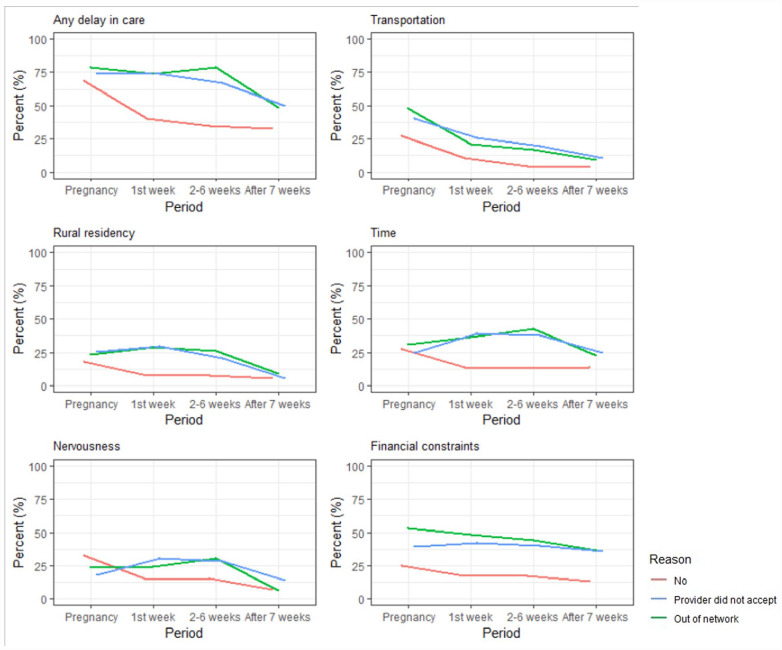
Relationship between insurance coverage denial and a delay in care by each reason.

In the assessment of the scope of any delay in care (Figure 1), delayed care was relatively high during pregnancy (72.7%), and then declined gradually in postpartum at 1 week (50.3%), 2 to 6 weeks (46.1%), and after 7 weeks (37.6%). The frequency and percentage of delayed care for a specific reason overall showed decreasing trends over time (Figure 2 and Table 2). However, a delay in care related to financial and time issues remained high across periods. Those who did experience insurance coverage denial showed a greater percentage of delayed care than those who did not experience insurance denial.

**Table 2.** Number of Participants for Delays in Care by Each Reason.

Reasons	Pregnancy (*n* = 586)	Postpartum (*n* = 527)		
		1 week	2–6 weeks	After 7 weeks
Any delay	426	265	243	198
Transportation	215	78	46	31
Rural residency	124	74	63	31
Time	162	110	111	88
Nervousness	153	97	102	42
Financial constraints	215	135	129	106

*Note.* Number includes multiple reasons reported by an individual participant.

Multivariable analysis revealed that individuals with providers that did not accept their insurance (OR = 1.74, 95% CI [1.04, 2.96], *p* = .037) had greater odds of a delay in care during pregnancy compared to those with no insurance problems (Table 3). In the first week of postpartum, those with insurance unaccepted by providers and those with insurance out of network had 3.37 times (95% CI: [1.81–6.53], *p* ≤ .001) and 3.47 times (95% CI: [2.00–6.18], *p* ≤ .001) greater odds of having a delay in care, respectively, compared to those with no insurance issues. These associations continued in 2 to 6 weeks postpartum, showing greater odds of having a delay in care by 5.74 times (95% CI: [3.01–11.58], *p* ≤ .001) for those with unaccepted insurance and 2.97 times (95% CI: [1.74–5.13], *p* ≤ .001) for those with insurance out of network, respectively. However, after 7 weeks postpartum, the figures were 1.54 times (95% CI: [0.86–2.75], *p* = .145) and 1.74 times (95% CI: [1.04–2.91], *p* = .036), respectively, showing diminished associations.

**Table 3.** Association Between Insurance Coverage Denial and a Delay in Care.

Factors	OR [95% CI]			
	Pregnancy (*n* = 586)	Postpartum (*n* = 527)		
		1 week	2–6 weeks	After 7 weeks
Insurance denial				
No	Ref.	Ref.	Ref.	Ref.
Provider did not accept	1.74 [1.04–2.96]*	3.37 [1.81–6.53]***	5.74 [3.01–11.58]***	1.54 [0.86–2.75]
Out of network	1.31 [0.78–2.21]	3.47 [2.00–6.18]***	2.97 [1.74–5.13]***	1.74 [1.04–2.91]*
Age (years)				
18–24	Ref.	Ref.	Ref.	Ref.
25–35	1.15 [0.62–2.09]	0.87 [0.48–1.58]	0.69 [0.38–1.24]	0.95 [0.53–1.71]
36–44	0.98 [0.49–1.92]	1.07 [0.56–2.09]	1.01 [0.52–1.97]	1.21 [0.64–2.32]
≥45	1.37 [0.51–3.90]	0.90 [0.43–1.87]	0.81 [0.39–1.69]	1.13 [0.55–2.31]
Race				
Non-Hispanic White	Ref.	Ref.	Ref.	Ref.
Non-Hispanic Black	1.72 [0.87–3.57]	1.33 [0.76–2.33]	1.53 [0.87–2.69]	1.64 [0.95–2.80]
Hispanic	0.69 [0.26–1.90]	1.79 [0.91–4.09]	1.62 [0.79–2.40]	0.97 [0.48–1.96]
Non-Hispanic Asian/Pacific Islander	0.97 [0.35–2.96]	0.79 [0.32–1.88]	0.84 [0.33–2.06]	1.03 [0.40–2.46]
Non-Hispanic Other	1.09 [0.68–1.75]	1.03 [0.59–1.77]	1.02 [0.59–1.78]	1.31 [0.76–2.45]
Insurance				
Employer based	Ref.	Ref.	Ref.	Ref.
Private pay	1.00 [0.54–1.89]	1.75 [0.95–3.27]	1.64 [0.90–3.03]	1.43 [0.80–0.26]
Medicaid	0.86 [0.48–1.54]	1.00 [0.56–1.79]	1.07 [0.60–1.92]	0.91 [0.51–1.62]
Medicare	0.92 [0.53–1.63]	1.44 [0.77–2.69]	1.50 [0.81–2.80]	1.54 [0.85–2.78]
Other	1.87 [0.43–13.1]	1.66 [0.66–4.29]	1.07 [0.41–2.78]	1.18 [0.48–2.85]
Marital status				
Never married	Ref.	Ref.	Ref.	Ref.
Married	0.77 [0.35–1.76]	1.13 [0.57–2.25]	0.92 [0.45–1.86]	1.70 [0.87–2.35]
Singled	0.97 [0.54–1.72]	1.21 [0.73–2.01]	1.49 [0.89–2.45]	1.44 [0.88–2.38]
Employment				
Unemployed	Ref.	Ref.	Ref.	Ref.
Retired/student/housewife	1.53 [0.56–4.30]	0.76 [0.36–1.61]	0.77 [0.51–1.68]	1.07 [0.50–2.29]
Part time	1.16 [0.53–2.51]	1.37 [0.67–2.82]	1.69 [0.82–3.52]	1.54 [0.75–2.23]
Full time	1.52 [0.72–3.17]	2.17 [1.13–4.23]*	1.87 [0.97–3.69]	1.70 [0.88–2.36]
Education				
Some high school	Ref.	Ref.	Ref.	Ref.
High school diploma	0.43 [0.06–1.77]	1.91 [0.72–5.35]	1.44 [0.54–4.07]	1.09 [0.43–2.92]
Some college	0.48 [0.07–2.02]	1.23 [0.45–3.55]	1.47 [0.54–4.27]	0.65 [0.24–1.81]
Associate degree	0.62 [0.09–2.72]	2.06 [0.71–6.31]	1.52 [0.52–4.66]	1.09 [0.39–3.16]
Bachelor’s degree	0.28 [0.39–1.17]	1.91 [0.68–5.70]	1.69 [0.59–5.05]	0.98 [0.36–2.77]
Graduate	0.13 [0.02–0.58]*	1.93 [0.66–5.89]	1.57 [0.54–4.81]	1.37 [0.49–3.94]
Live births				
0	Ref.	Ref.	Ref.	Ref.
1	0.77 [0.42–1.42]	0.48 [0.20–1.13]	0.56 [0.23–1.31]	0.52 [0.23–1.18]
2	0.57 [0.29–1.11]	0.58 [0.23–1.45]	0.76 [0.31–1.88]	0.45 [0.19–1.06]
≥3	0.39 [020–0.75]***	0.48 [0.19–1.19]	0.53 [0.21–1.30]	0.40 [0.17–0.95]*

*Note.* ORs = odds ratios; CI = confidence intervals.

Statistical significance * < .05, ** < .01, *** < .001.

**Figure 3.**
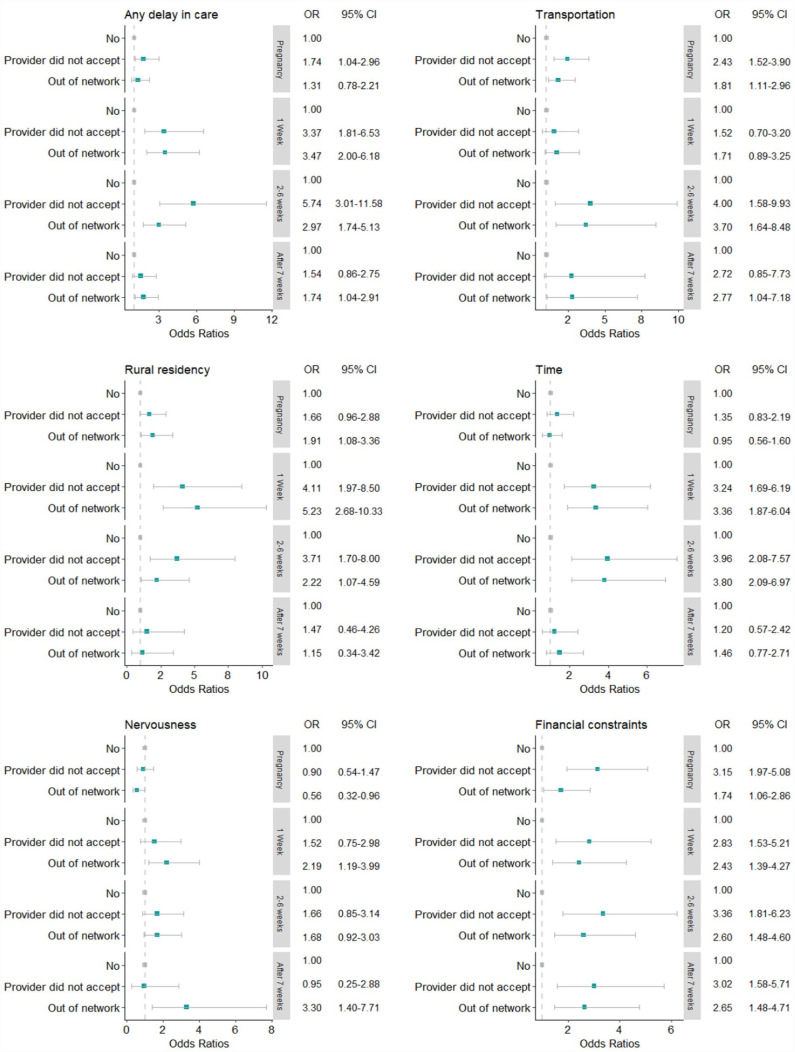
Association between insurance coverage denial and a delay in care characterized by reason.

Figure 3 depicts the ORs for specific reasons for delays in care at each time point. Transportation showed significant ORs during the 2 to 6 weeks postpartum and the ORs diminished afterward. Rural residency and time issues also showed significant ORs during the 1 week and 2 to 6 weeks postpartum, but ORs shrank after 7 weeks postpartum. In contrast, financial constraints showed significant ORs to remain consistent throughout the whole period, while nervousness about seeing a doctor did not have significant associations over the study period.

## Discussion

Our study investigated the association of insurance coverage denial with a delay in care during pregnancy and the postpartum period using nationwide survey data in 2022. Our study provides important evidence for healthcare experience at various time points, 1 week, 2 to 6 weeks, and after 7 weeks postpartum. Approximately 72.7% of participants reported a delay in care during pregnancy, and the percentage declined over time during the postpartum period: first week 50.3%, 2 to 6 weeks 46.1%, and after 7 weeks 37.6%, respectively. In multivariable models, those with insurance coverage denial faced an intensified delay in care throughout the pregnancy trajectory compared to women without insurance problems. Notably, in the analysis of a delay in care for specific reasons, the association of insurance coverage denial with a delay in care encapsulated transportation, rural residency, and time issues during the first 6 weeks postpartum but diminished afterward. In contrast, financial constraints tended to sustain the association between insurance coverage denial and an increased delay in care.

### Associations Between Insurance Coverage Denial and a Delay in Care

Our analysis showed that individuals who experienced insurance coverage denial reported amplified challenges to accessing care both during pregnancy and postpartum. These associations are consistent with previous reports that insurance issues were associated with delays in care in general (Wallace et al., 2020; Wilcox et al., 2016). The lingering associations of insurance coverage denial on delaying care during and after pregnancy were noteworthy. However, there were observed variations that the associations were particularly noticeable in the first week (3.4–3.5 times higher odds) and 2 to 6 weeks (3.0–5.7 times higher odds) postpartum. While postpartum care commonly takes place around 6 weeks after childbirth, previous studies note that many postpartum health problems, such as severe hypertension, severe bleeding, and postpartum depressive symptoms, occur as early as 1 week after delivery (Agency for Healthcare Research and Quality, 2022; Bauman et al., 2020; Florio et al., 2021; Lopez-Gonzalez & Kopparapu, 2022). These clinical aspects suggest that timely and adequate postpartum care is critical for women to recover from delivery and to ensure their physiological and psychosocial well-being in the long run (Cheng et al., 2006; Salam et al, 2014; Shaw et al., 2006). Postponed or delayed postpartum care may further introduce or aggravate health complications and increase preventable emergency visits or hospitalizations (Brousseau et al., 2018; Callaghan et al., 2012). Current results indicate that despite the clinical significance of postpartum care, those having insurance issues may face additional difficulties in obtaining care during the early postpartum period, which raises significant concerns about maternal health after delivery and provides practical implications for proactive postpartum management (The American College of Obstetrician and Gynecologists, 2018).

### Specific Reasons for a Delay in Care

Although the overall association of insurance issues with a delay in care is highlighted, understanding delayed care typified by specific reasons is invaluable for further practical implications. We found that when insurance was denied, delayed care resulted from several reasons including a lack of transportation, rural residency, and a lack of time. Previous studies have identified these issues as important barriers for limited access to care during pregnancy through the first 6 weeks postpartum (Altman et al., 2020; Heaman et al., 2015; Interrante et al., 2022). Although future longitudinal research is needed, one explanation for this finding may be that women perceive these types of delays in care, coupled with their clinical needs, as pressing in the early postpartum period, whereas these barriers are less consequential after 7 weeks postpartum (Bloch et al., 2018; Heaman et al., 2015; Kitsantas et al., 2012; Webb et al., 2014).

For a delay in care attributed to financial constraints, insurance coverage denial revealed sustained associations over time. The magnitude of the estimated associations remains fairly constant over the pregnancy and the whole postpartum period, suggesting insurance coverage denial has lasting impacts on delayed care. Financial problems are often referred to as major barriers to accessing care for those uninsured and even for those insured (Loewenstein et al., 2013). The present study supports prior work showing that in the face of the insurance coverage denial, individuals suffer a significant delay in care related to financial constraints over an extended period (Loewenstein et al., 2013; Taylor et al., 2021). Future studies to investigate a delay in care with respect to financial aspects are warranted to improve our understanding of these observed associations.

As our study was conducted during the ongoing COVID-19 pandemic, the disrupted health care may have shaped a landscape in which diminished capacity or closures of clinics or hospitals in communities possibly posed barriers to accessing perinatal care (Anderson et al, 2021; Czeisler et al, 2020). Furthermore, individual factors, such as fear of exposure to the novel virus, increased family responsibilities, and financial instability, could have also impacted individuals’ ability to seek care (Anderson et al, 2021; Lee, 2023; Power, 2020). While our findings may complement previous reports on increased barriers in the unique healthcare context during the COVID-19 pandemic, caution should be in place when interpreting our findings as the current study did not specifically examine the impact of the COVID-19 pandemic on delays in care.

### Practice, Public Health, and Policy Implications

The United States has made significant improvements in access to care by reducing the uninsured rate through national health reform, which enhanced access to care and disease management in various health areas, including prenatal and postpartum care (Adams et al., 2019; Eliason et al., 2021; S. H. Gordon et al., 2020). Importantly, Medicaid accounts for approximately 40% of births in the United States, making it an important source of coverage for many women (Corallo et al., 2022). However, individuals who exceed the income threshold are not eligible for Medicaid after the 60-day postpartum period. Some enrollees lose coverage due to administrative barriers in the renewal process. This gap is particularly concerning for women living in states that have not expanded Medicaid under the ACA. Recently, the Consolidated Appropriations Act (CAA) of 2023 allowed states to expand coverage to 12 months postpartum, and as of April 2023, 31 states and the District of Columbia have opted for this extension (S. Gordon et al., 2023). Previous reports suggest that approximately 1.5 million people would have benefited if all states extended postpartum coverage to 12 months in 2021 (Corallo et al., 2022; S. Gordon et al., 2023). Markedly, many pregnancy-related health conditions occur after childbirth and require ongoing monitoring far beyond the 60-day postpartum period. These highlight the importance of providing continuous coverage to meet the critical postpartum care need and improve maternal health outcomes. States that have not implemented the Medicaid coverage expansion should consider doing so to address the healthcare gap among new mothers.

Notably, women, infants, and children (WIC) program, which serves millions of pregnant and postpartum, provides referrals to health insurance and medical care with evidence showing it is effective for supporting healthy pregnancies (Carlson & Neuberger, 2021). Yet, a family must have gross income of no more than 185% of the federal poverty level ($40,182 for a family of three as an example) and be at nutritional risk in order to participate. However, researchers have identified a significant gap between those who qualify and those who are enrolled in WIC (Hall & Neuberger, 2021). Investing in community-based outreach efforts to enroll women in WIC and provide health education on health insurance is an important next step to closing the gap in health insurance-related issues in delayed care during the prenatal and postpartum periods.

Insurance denials occur in various types of insurance coverage for a variety of reasons, such as inadequate coverage for preventive services, reimbursement issues, prior authorization process, and limited understanding of insurance policies, including coverage restrictions and out-of-network providers (American Medical Association, 2018; Levy & Janke, 2016). While these factors are noteworthy, social stigma may also contribute to insurance-based bias and insurance denials (Decker, 2011; Han et al., 2015). It is crucial to clearly communicate the health services covered under the insurance policy and ensure beneficiaries understand their policies, while healthcare professionals increase awareness of potential insurance-based prejudice in clinical settings. Since its enactment, the ACA has significantly reduced the uninsured rate and improved access to care. However, policymakers should continue to work collaboratively with healthcare stakeholders to address practical barriers to accessing care from a holistic perspective.

Our findings provide important implications for the healthcare community and policymakers to implement methods to reduce the barriers to accessing care during and after pregnancy. This is especially important as policymakers and lay persons discuss the possibility of implementing a universal health care (UHC) plan. While UHC in most countries usually includes a set of basic preventive and reproductive health services including access to care for pregnant women before, during, and after birth (Quick et al., 2014), inequities persist as UHC programs are implemented with evidence of inverse care laws emerging—whereby those who are most in need for care are the least likely to receive it. As such, simply providing insurance may not be sufficient to promote population health. In fact, the findings of our study demonstrate that being insured itself does not guarantee improved access to pregnancy-related health care. Nearly half of the individuals experienced an insurance coverage denial, and more than half of the women reported a delay in care during or after pregnancy. Importantly, the association between delay in care and insurance coverage denial shows various patterns over the course of pregnancy across specific reasons of delayed care. To remediate such issues, policymakers should also consider funding initiatives to build infrastructure, resources to strengthen healthcare capacity, and strategies to promote health equity in delivering basic maternal health services.

The strength of the study is that it is a national sample, with quotas for ensuring racial/ethnic representation similar to the total U.S. population of birth mothers. However, this study is not without limitations. First, despite our efforts to increase the response rate by incentivizing participation, approximately 46.7% of the survey response rate is considered low and indicates a limitation. Second, as a pooled cross-sectional study, we cannot draw cause and effect conclusions about associations between insurance issues and delayed care. Third, despite accounting for identified covariates, unmeasured confounding from other factors could still be present. Fourth, there may be other reasons for insurance coverage denial or delayed care we did not consider, and this may lead to an underestimation of the true association between these variables. Fifth, our study analyzed population-based survey data. While the findings from this unique dataset advance the evidence base of the association between health insurance denial and delays in care, we did not examine this association in clinical settings. Future research is warranted to investigate this relationship using clinical data to confirm or strengthen our findings. Lastly, due to the self-report nature of data collection, there is a possibility of recall and social desirability bias. In addition, we did not ask respondents to identify delays in care by trimester during pregnancy, which would have provided additional data. This is another important consideration for future studies.

## Conclusions

In the United States, more than half of all maternal deaths occur in the postpartum period (Baeva et al., 2018), and despite a wealth of evidence showing such deaths can be avoided with early detection and treatment, we found a significant proportion of women in this sample experienced delayed care throughout the prenatal and postpartum period. Insurance coverage denial and other financial issues are associated with delays in care during the pregnancy and postpartum periods. As a means of reducing delays in care, practitioners should assess structural barriers including lack of transportation, rural residency, and a lack of time due to employment and childcare constraints. Strategic policy interventions targeted at various barriers could reduce delayed care and help achieve effective prenatal and postpartum health for mothers and their newborns.
